# Society Meetings

**Published:** 1905-12

**Authors:** 


### SOCIETY MEETINGS.

The Medical Society of the County of Erie will hold its eighty-fifth annual meeting Tuesday, January 9, 1906, at 10 o'clock a. m. in the rooms of the Society of Natural Sciences, Buffalo Library Building. John D. McPherson is president; F. C. Gram is the secretary.

---

The Semi-Annual Meeting of the Medical Society of the County of Chautauqua, was held at Dunkirk, December 13, 1905. Program: Business Session 11 a. m. Scientific Session 1:30 p. m. Vice-president's Annual Address, Dr. A. W. Dods, Fredonia; Arteriosclerosis, Dr. J. A. Weidman, Dunkirk; The Medical Man, Dr. J. J. Sharp, Silver Creek; Some Practical Points on the Finer Diagnostic Methods, Dr. A. E. Woehnert, of Buffalo. C. H. Richards, president; C. A. Ellis, secretary.

---

The Erie County Medical Association held its regular meeting Monday, December 11, 1905, at 8:30 p. m., at the University Club. Program: I. Dermatological Anomalies and Curiosities,

Grover W. Wende, (Illustrated with stereopticon). II. Nephroptosis, Julius Ullman. Arthur G. Bennett, president; D. E. Wheeler, secretary, 564 Delaware Ave., Buffalo.

---

THE Buffalo Academy of Medicine held meetings during the month of November as follows:

*Section on Surgery.*—Wednesday, November 8, 1905, at 8:30 P. M. Program: (a) Massage of the heart in cases of impending death, William C. Phelps; (b) Traction as applied to tubercular joint conditions, Prescott LeBreton.

*Section of Medicine.*—Tuesday, November 14, 1905 at 8:30 P. M. Program: (a) The prognosis and treatment of chronic valvular disease of the heart, DeLancey Rochester, discussion opened by Allan A. Jones; (b) Nephritis, Cornelius J. Carr; discussion opened by Thomas B. Carpenter.

*Section of Pathology.*— Tuesday, November 21, 1905, at 8:30 P. M. Program: (a) Present status of our knowledge concerning digestion, Frederick C. Busch; (b) Report of a case of fatal prostatism in a cryptorchid, with specimen, David E. Wheeler.

*Section of Obstetrics and Gynecology.*—Tuesday, November 28, 1905, at 8:30 P. M. Program: Ectopic gestation, Carlton C. Frederick.

---

THE Esculapian Club recently elected officers for the current year as follows: president, Dr. C. J. Reynolds; vice-president, Dr. F. J. Carr; secretary, Dr. Fred S. Hoffman. The club holds meetings on the third Thursday evening of each month.

---

THE Southern Surgical and Gyneological Association held a splendid meeting at Louisville, December 12-14, 1905, under the presidency of Dr. Lewis C. Bosher of Richmond. The meeting was atended by a large number of surgeons from all parts of the country and its scientific work was of the best. The banquet on the evening of the 13th was of the first order. Dr. Charles A. L. Reed of Cincinnati, acted as toastmaster and introduced the speakers with original touches of humorous mélange. Those who responded to sentiments were: Dr. Joseph M. Mathews, of Louisville; Dr. Henry T. Byford, of Chicago; Dr. Robert T. Morris, of New York, Dr. Robert S. Hill, of Montgomery, Ala.; and Dr. Charles H. Mayo, of Rochester, Minn.

The next place of meeting is Baltimore, under the following administration: president, G. H. Noble, of Atlanta; vice-presidents, Stuart McGuire, of Richmond, and E. D. Martin, of New Orleans; secretary, W. D. Haggard, of Nashville, re-elected; treasurer, Charles M. Rosser, of Dallas, Texas, re-elected. Dr. Lewis S. McMurtry, of Louisville, was re-elected chairman of the council; and Dr. Howard A. Kelly, of Baltimore, was elected chairman of the local committee of arrangements.

---

THE Buffalo Association of Columbia Medical Alumni was organised November 2, 1905, at which time it held its first dinner. The officers elected were: president, Dr. Carlton R. Jewett; vice-president, Dr. Arthur W. Hurd; secretary, Dr. E. L. Bebee. It is purely a social organisation and is to meet twice a year, once about March first, and again at or near November first.

